# Effects of Hydrothermal and Microwave Dual Treatment and Zein on the Enzymolysis of High Amylose Corn Starch

**DOI:** 10.3390/gels8010029

**Published:** 2022-01-04

**Authors:** Jie Liu, Qiuye Yang, Tiantian Yuan, Yawei Liu, Guihong Fang

**Affiliations:** 1 College of Food Science and Engineering, Henan University of Technology, Zhengzhou 450001, China; liujie@haut.edu.cn (J.L.); 15904927191@163.com (Q.Y.); yuantiantian0226@163.com (T.Y.); 2 Department of Nutrition and Food Hygiene, Hainan Medical University, Haikou 571199, China; 3 School of Food Science and Engineering, South China University of Technology, Guangzhou 510640, China

**Keywords:** high-amylose corn starch, hydrothermal treatment, microwave treatment, zein, enzymolysis, resistant starch

## Abstract

Resistant starch (RS) type 2-high-amylose corn starch (HACS) was subjected to simultaneous hydrothermal (25% moisture content, 90 °C for 12 h) and microwave (35% moisture content, 40 W/g microwaving for 4 min) treatment and zein (at a zein to treated starch ratio of 1:5, 50 °C for 1 h) to improve its resistance to enzymolysis. Scanning electron microscopy (SEM) highlighted the aggregation and adhesion of the composite. The average particle size of the composite (27.65 μm) was exceeded that of both the HACS (12.52 μm) and the hydrothermal and microwave treated HACS (hydro-micro-HACS) (12.68 μm). The X-ray diffraction results revealed that the hydro-micro-HACS and composite remained B-type, while their crystallinity significantly decreased to 16.98% and 12.11%, respectively. The viscosity of the hydro-micro-HACS and composite at 50 °C was 25.41% and 35.36% lower than that of HACS. The differential scanning calorimetry (DSC) results demonstrated that the composite displayed a new endothermic peak at 95.79 °C, while the weight loss rate and decomposition temperature were 7.61% and 2.39% lower than HACS, respectively. The RS content in HACS, the hydro-micro-HACS, and composite was 47.12%, 57.28%, and 62.74%, respectively. In conclusion, hydrothermal and microwave treatment combined with zein provide an efficient physical strategy to enhance the RS type 2-HACS.

## 1. Introduction

The increase in consumer demand for healthy food products has led to new food processing and composition requirements. The protein–starch composite can be used as biological material in the food industry [1], the medical field, environmental protection, and textiles [2]. The protein–starch composite displays unique processing, anti-oxidative and anti-digestive properties, as well as other functional characteristics as food additives and ingredients [3].

Although resistant starch (RS), also known as anti-enzymatic starch, cannot be digested and absorbed in the small intestine, it can be fermented by the microbial flora in the colon. It presents beneficial physiological effects [4], such as regulating blood sugar levels, significantly improving intestinal health, preventing intestinal disease and colon cancer [5], and reducing cholesterol levels. RS is also considered a component of dietary fiber. Compared with traditional dietary fiber, RS presents advantages, such as a delicate taste, low water holding capacity, and low calorific content. Therefore, it is widely used in bread, tortillas, snack products, and fried foods to lower the glycemic index and as a food thickener in sauces, soups, and beverages without affecting the taste and flavor [6]. Furthermore, RS is classified into four types according to its resistance to enzymatic hydrolysis [7]. The starch granules of RS1 are wrapped by cell walls and protein [8]. RS2 displays a dense molecular structure of natural starch granules, making enzymes inaccessible. The RS in RS3 is formed via starch retrogradation after gelatinization. RS4 represents chemically modified starches, such as ethers and esters. Additionally, RS5 denotes a starch–lipid V-type complex [9]. As a high amylose corn starch (HACS), RS2 does not gelatinize during cooking [10]. Hydrothermal and microwave treatment represent two effective methods to increase RS content [11]. Storing starch at a low temperature (−20 °C) after hydrothermal treatment can significantly increase the RS content in retrograded products. Zhang et al. indicated that the RS content of canna starch doubled at a 20% moisture content level and a microwave power of 1000 W for 30 min [12]. Son Trinh Khanh et al. revealed that the slowly digestible starch (SDS) content of starch treated at a 30% moisture content level for 24 h reached 49.1%, which was 31.9% higher than in the control group after the hydrothermal treatment of water yam starch [13]. Molavi et al. reported that hydrothermal treatment increased starch solubility and gelatinization temperatures, especially the onset temperature (To), but decreased swelling power and amylose leaching, relative crystallinity, gelatinization enthalpy, and gelatinization range [14]. Andrade reported similar results after subjecting cassava starch to hydrothermal treatment [15]. Li et al. reported that microwave-toughening treatment increased the amylose and RS content of potato starch from 26.08% and 11.54% to 35.06% and 27.09%, respectively [16]. Fan et al. found that the crystallinity of microwave-treated rice and potato starch decreased to some extent while displaying lower amorphous layer thickness and increased crystalline layer thickness [17]. Wei et al. reported that microwave and hydrothermal treatment decreased the solubility and swelling power of water caltrop starch. The water caltrop starch presented a C-type crystalline pattern which changed into an A-type pattern after microwave treatment. After 50 s of microwave treatment, this starch displayed the highest amylose (48.78%), crystallinity (42.95%), and RS content (67.41%, uncooked and 48.91%, cooked) [18]. Zailanithe et al. showed that microwave-modified sago (Metroxylon sagu) starch exhibited higher RS levels [19].

Zein is a yellow powder belonging to a class of prolamins and is applied in coatings, films, fibers, plastics, adhesives, and inks [20]. Zein can encapsulate starch granules after freeze-drying and low-temperature spray drying to increase their anti-digestive properties [21]. Combining proso millet with zein decreased the rapidly digestible starch (RDS) content while increasing the SDS and RS levels [22]. Moreover, hydrothermal treatment, corn oil, and zein significantly increased the SDS and RS content in the composite [23]. Gao et al. indicated that the gelatinization temperature and final viscosity value of a waxy corn starch and zein composite decreased, while the storage modulus value, enthalpy value, and thermal stability increased [24]. Zein may positively impact the rheological properties of dough and consequently the development of strain hardening behavior [25]. Studies have shown that the enzymatic hydrolysis of zein can change the nutritional and functional properties of food proteins, such as improving digestibility, modifying sensory quality (such as texture or taste), enhancing antioxidant activity, and reducing allergenicity [26]. However, limited information is available regarding the impact of physical treatment and zein on the RS content. This study investigates the modification effect of combining hydrothermal, microwave, and zein treatments on the enzymolysis of high-amylose corn starch, providing an alternative method for RS preparation.

## 2. Results and Discussion

### 2.1. Morphological Characteristics

**Figure 1:**
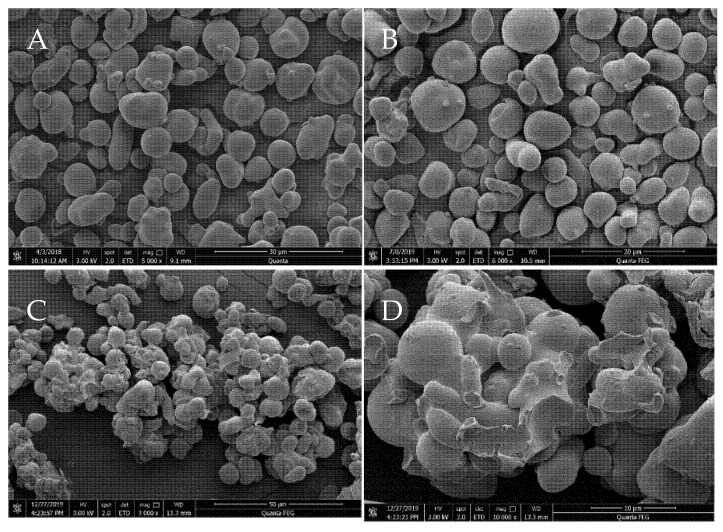
Scanning electron micrographs of the HACS ((**A**): 5000× magnification), hydro-micro-HACS ((**B**): 6000× magnification), and the composite ((**C**,**D**): 3000× and 10,000× magnification).

HACS granules are mostly round or oval, while a few are rod-shaped with smooth surfaces (Figure 1A). The high-amylose corn starch granules were independent of each other and showed no adhesion after hydrothermal and microwave treatment (Figure 1B). Furthermore, the granule morphology was not modified, which was consistent with the results observed by Vamadevan et al. who subjected corn starch granules to hydrothermal treatment [27]. Zhong et al. found that no changes were evident in the size and shape of the HACS after microwave treatment [28], while the zein and treated starch composite showed agglomeration. The starch granules were wrapped by a continuous phase formed by zein (Figure 1C, ×3000, Figure 1D, ×10,000). In a previous study, a composite was prepared consisting of hydroxypropyl tapioca starch and zein. The results showed that an increased zein level or reaction time caused the starch granules to adhere to or be wrapped by the zein and were even embedded in the zein matrix [29]. Leroy et al. observed the dispersion of large starch aggregates into a continuous zein matrix at a zein content level exceeding 50% [30]. The zein was aggregated and distributed on the surface of the starch granules due to its precipitation after adding cold water. Therefore, the starch granules were protected, inhibiting the enzymatic action.

### 2.2. Color

No significant differences were evident between the redness (a*) values of the three samples (*p* > 0.05) (Table 1). The HACS presented higher yellowness (b*) values, while lower L* values were evident in the hydro-micro-HACS (*p* < 0.05) (Table 1). This experiment used commercial yellow zein containing a high xanthophyll pigment concentration (8–9%), consequently increasing the b* values and decreasing the lightness (L*) values of the composite. The konjac glucomannan and zein composite gel presented a yellow color [31].

### 2.3. Particle Size

The average particle sizes in HACS, hydro-micro-HACS, and the composite were 12.52, 12.68, and 27.65 μm, respectively (Table 1). Hydrothermal and microwave treatment did not significantly change the particle sizes of HACS (*p* > 0.05). Luo et al. reported that microwave treatment did not alter the sizes and shapes of the granules in normal maize, waxy maize, and amylomaize V starches [32], while those of the composite increased significantly (*p* < 0.05), which was consistent with the scanning electron micrographs. The encapsulation of the protein around starch granules causes agglomeration, increasing their average particle sizes [29]. 

### 2.4. X-ray Diffraction

**Figure 2:**
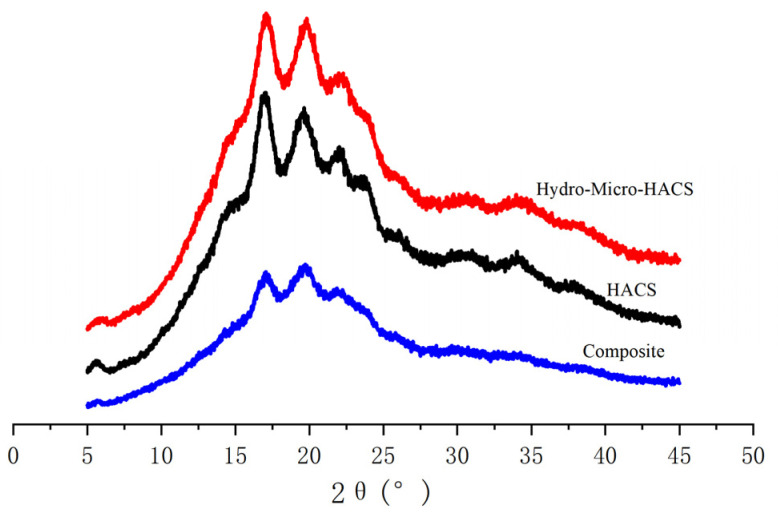
The X-diffraction patterns of the HACS, hydro-micro-HACS, and composite. Different letters following the crystallinity value indicate significant differences (*p* < 0.05).

The X-ray diffraction patterns of HACS, hydro-micro-HACS, and composite presented diffraction peaks at 2θ of 5.6°, 17°, 19.5°, 22°, and 24° (Figure 2). These peaks were characteristic of a B-type X-ray pattern, indicating that the hydrothermal and microwave treatment and the addition of zein did not change the crystalline structure in high-amylose corn starch. The absorption intensity of the diffraction peaks decreased after dual treatments (16.98% crystallinity) and further zein addition (12.11% crystallinity) (Table 1). Molavi et al. and Pinto et al. reported that although hydrothermal treatment reduced the relative crystallinity of acorn starch, it did not alter the X-ray diffraction pattern [14,33]. Furthermore, hydrogen bond breakage during hydrothermal treatment caused starch crystallite disruption [11]. Yin et al. and Ma et al. showed that microwave treatment decreased the crystallinity of laird lentil starch [34,35]. These results indicated that microwave treatment disrupted the intra- and inter-molecular hydrogen bonding in starch, damaging the double-helical structure and destroying the crystalline arrangement [36]. Furthermore, the zein partially replaced the starch in the complex, weakening the absorption peak intensity.

### 2.5. Viscosity

The HACS, hydro-micro-HACS, and composite viscosity were 362, 270, and 234 cP, respectively. An increase in the viscosity of the paste during the heating phase of gelatinization indicated the water absorption level and swelling capacity of the starch [37], while a viscosity increase during the cooling phase was attributed to the hydrogen bond aggregation of the amylose [38]. Pinto et al. indicated that hydrothermally treated starch showed lower peak viscosity than the native starch [33]. The viscosity peak, viscosity at holding (95 °C) and cooling periods (50 °C), setback, and consistency of canna starch decreased compared to its native starch counterpart [39]. Hydrothermal and microwave treatment reduced the viscosity of HACS at 50 °C, indicating a lower tendency for retrogradation [40]. Mutlu et al. [41] demonstrated that microwave treatment caused low cold viscosity. The composite exhibited the lowest viscosity of the three samples, indicating that zein further inhibited starch molecule retrogradation.

### 2.6. Thermal Properties

The weight loss rate of the HACS, hydro-micro-HACS, and composite was 80.99%, 80.29%, and 74.83% (Table 1), respectively, while no significant differences were evident between HACS and hydro-micro-HACS (*p* > 0.05). The significant decrease in the weight loss rate of the composite may be due to the zein and starch combination. At maximum HACS, hydro-micro-HACS, and composite decomposition rates, the decomposition temperatures were 316.98, 317.84, and 309.41 °C, respectively. The increased decomposition temperature during the dual treatments (*p* < 0.05) indicated enhanced thermal stability, while the internal molecule structure became firmer. The decreased decomposition temperature of the composite may be caused by the partial replacement of the starch by zein.

The hydro-micro-HACS showed a significant increase in the To and peak gelatinization temperature (Tp) compared to HACS (*p* < 0.05) (Table 2). Dual treatment significantly decreased (*p* < 0.05) the energy required for gelatinization (∆H). The subsequent increase in the To and Tp and decrease in ∆H were consistent with the modification results obtained in waxy maize starch via hydrothermal treatment and crosslinking [42]. Hydrothermal treatment led to a perfect and less crystalline structure [43]. Molavi et al. reported that increased gelatinization temperatures might be related to amylose-amylose and amylose–amylopectin interaction since the amylose leaching decreased significantly (*p* < 0.05) after hydrothermal treatment [14]. The composite presented two peaks after zein addition. The first endothermic peak occurred at a To of 84.77 °C and a completion gelatinization temperature (Tc) of 104.97 °C, while the second endothermic peak was evident between a To of 134.76 °C and a Tc of 138.62 °C (Table 2). The temperatures of the second endothermic peak of the composite were the same as the treated starch (*p* > 0.05), while the ∆H decreased significantly (*p* < 0.05). The decreased ∆H could be attributed to the partial replacement of the treated starch by zein. Therefore, the starch concentration was reduced, and less energy was required to destroy the crystalline structure during gelatinization [44].

### 2.7. Enzymolysis

The RS levels in the samples were used to characterize the degree of enzymatic hydrolysis. The RS content in HACS, hydro-micro-HACS, and composite was 47.12%, 57.28%, and 62.74%, respectively. The hydrothermal and microwave treatment and compounded zein increased the RS by 21.56% and 33.15%, respectively. Brumovsky and Thompson found that hydro-micro-HACS displayed a resistance level of 43.9% compared to native starch at 18.4% [45]. Zhong et al. indicated that HACS subjected to microwave treatment for 1 min displayed a lower degree of hydrolysis than the native sample [28]. Sun et al. speculated it might be challenging for microwave-treated samples to make contact with digestive enzymes, decreasing the hydrolysis rate [46]. The perfect crystalline structure resulting from hydrothermal and microwave treatment increased the gelatinization difficulty and enzymatic hydrolysis of RS 2 [42]. The presence of zein in the composite further hampered gelatinization and hydrolysis from its spatial barrier to the enzyme. Xu et al. showed that the physical barrier of the zein matrix restricted water ingress, heat transfer, and adequate space for granule swelling, consequently preventing the enzyme access to the starch [21]. Kljak et al. reported that the starch digestibility rate decreased when zein was present in the starch. When starch granules were embedded in a complex matrix, zein limited their accessibility to enzymes, influencing the starch digestibility rate [47].

## 3. Conclusions

Hydrothermal and microwave treatment and zein increase the RS content in HACS by 21.56% and 33.15%, respectively. Hydrothermal and microwave treatment decreases the crystallinity, viscosity, and ∆H of HACS by 16.98%, 25.41%, and 49.18%, respectively. The zein encapsulation of the modified starch granules increases the average particle sizes and b* value of the composite by 118% and 124%. The crystallinity, viscosity, lightness, weight loss rate, and decomposition temperature of the composite decrease by 28.68%, 13.33%, 3.02%, 6.80%, and 2.65%, respectively. The results reveal that hydrothermal and microwave treatment perfected the crystalline structure of the starch granules, while zein encapsulation facilitates enzymatic hydrolysis. Therefore, developing a new method for RS preparation is essential, and further exploration of the application in various food matrices is required.

## 4. Materials and Methods

### 4.1. Materials

The HACS was supplied by Hengrui Technology Co., Ltd. (Luohe, China). The RS test kits were acquired from Ireland Megazyme Company, and zein was purchased from Sinopharm Chemical Reagent Co., Ltd. (Shanghai, China). The anhydrous ethanol was obtained from Tianli Chemical Reagent Co., Ltd. (Tianjin, China), while the sodium hydroxide was purchased from Tianjin Kemeo Chemical Reagent Co., Ltd. (Tianjin, China).

#### 4.1.1. Preparation of the Hydro-Micro HACS

Hydrothermal treatment occurred according to a method described by Sui et al. [48]. The moisture content of the HACS was adjusted to 25% with distilled water after equilibration at room temperature for 24 h. The sample was placed in a sealed high-temperature-resistant glass bottle and dried at 90 °C for 12 h, after which it was refrigerated at 4 °C for 10 h. The refrigerated HACS dried at 40 °C to a moisture content of 10%. 

After hydrothermal treatment, the moisture content of the HACS was adjusted to 35% with distilled water and equilibrated at room temperature for 24 h. The sample was microwaved at 40 W/g for 4 min and refrigerated at 4 °C for 10 h, after which it was dried at 40 °C to a moisture content of 10%. The samples were ground and sieved using an 80-mesh sieve for further analysis. 

#### 4.1.2. Preparation of the Zein and Hydro-Micro HACS Composite

Zein (6% *w*/*w*) was dissolved in ethanol (70%) and stirred in a water bath at 30 °C until completely dissolved. Then, hydro-micro-HACS (at a 1:5 ratio of zein to starch) was slowly added while rapidly stirring in a 50 °C water bath for 1 h. Next, cold water was added at 80 times the protein content, stirred quickly for 30 min, and allowed to settle. After removing the supernatant solution, the precipitate was dried at 45 °C, ground, and sieved using an 80-mesh sieve for further analysis. 

### 4.2. Scanning Electron Microscopy (SEM)

The HACS, hydro-micro-HACS, and composite surfaces were observed and recorded using SEM (Quanta250 FEG, FEI, Hillsboro, OR, USA) at an accelerating voltage of 3 kV in high-vacuum conditions.

### 4.3. Color

The color of the HACS, hydro-micro-HACS, and composite was measured using a Konica Minolta colorimeter CR-400 (Osaka, Japan) and the L*(the degree of lightness), a*(the red-green axis), and b* (the yellow-blue axis) values were recorded.

### 4.4. Particle Size

The HACS, hydro-micro-HACS, and composite particle size distributions were determined using a laser diffraction particle size analyzer (SALD-301V, SHIMADZU, Kyoto, Japan).

### 4.5. X-ray Diffraction

The X-ray diffraction patterns were determined using an X-ray diffractometer (Rigaku MiniFlex600, Tokyo, Japan) at a scanning speed of 4°/min, angles ranging from 5° to 45° (2θ), 40 kV, and 30 mA. The MDI Jade 6.0 software (Material Date, Inc., Livermore, CA, USA) was used to analyze the crystallinity of the samples.

### 4.6. Viscosity

The HACS, hydro-micro-HACS, and composite were mixed with distilled water to obtain slurries (6%, *w*/*v*), which were placed in separate sealed bottles. The samples were gelatinized in an autoclave at 126 °C for 30 min, while their viscosity levels were measured at 50 °C using a DV-Ⅲ rotary viscometer.

### 4.7. Thermal Properties

The HACS, hydro-micro-HACS, and composite thermal characteristics were measured using a thermogravimetric analyzer (TGA; Q50, TA, Instruments, New Castle, DE, USA) and differential scanning calorimeter (DSC; Q20, TA, New Castle, DE, USA). The sample (10 mg, db) was placed in a platinum pan and heated from 40 °C to 600 °C at a heating rate of 10 °C/min to obtain the weight loss and weight loss rate values. The composite (2.5 mg, db) was mixed with 7.5 μL in an aluminum pan, which was hermetically sealed and equilibrated at room temperature for 24 h. The sample and reference were scanned from 20 °C to 160 °C at a heating rate of 10 °C/min.

### 4.8. Enzymolysis

The HACS, hydro-micro-HACS, and composite enzymolysis were determined using a Megazyme RS detection kit [4]. The sample (100 mg) was incubated in a solution containing pancreatic amylase and amyloglucosidase in a water bath at 37 °C for 16 h, after which an equal volume of ethanol was added to terminate the reaction. The solution was centrifuged, and the supernatant was discarded, after which the precipitate was washed twice with ethanol. The precipitated flocs were placed in an ice water bath, dissolved by adding 2 M KOH, and stirred vigorously with a magnetic stirrer. The solution was adjusted to neutral with acetate buffer, and the starch was quantitatively hydrolyzed into glucose with Aufrecht Melcher Grossaspach (AMG), after which the glucose content of the sample was measured using a glucose oxidase-preoxidase (GOPOD)kit. The RS content was calculated as the free glucose product released via RS hydrolysis using amyloglucosidase with a correction factor of 0.9, RS = glucose × 0.9.

(1) $$w=\frac{E\times F\times 0.9}{W}\times 100\%$$

where *w* denotes the RS content (%), E is the absorbance difference between the sample and the blank reagent, F signifies the conversion from the absorbance value to the microgram—an absorbance value of 100 μg d-glucose was determined during the GOPOD reaction, F = 100 (the number of μg of d-glucose divided by the GOPOD absorbance value of 100 μg d-glucose), and W is the dry starch weight (g).

The RS levels were used to characterize the HACS, hydro-micro-HACS, and composite enzymolysis. The RS content in the composite was calibrated using the starch percentage in the composite and was calculated as

(2) $$\mathrm{RS}\;(\mathrm{actual}\;\mathrm{content})\%=\frac{\mathrm{RS}\;(\mathrm{measured}\;\mathrm{value})}{\mathrm{Starch}\;\mathrm{ratio}\;\mathrm{in}\;\mathrm{composite}}\times 100\%$$

### 4.9. Statistical Analysis

All measurements were performed in triplicate. The data were analyzed using SPSS Version 16.0. Duncan’s pairwise comparison was used to assess the differences between the mean values, and *p* < 0.05 was considered statistically significant based on the analysis of variance (ANOVA) test.

## Figures and Tables

**Table 1:** Color, particle sizes, viscosity, thermal gravimetric analyzer (TGA) characteristics, and crystallinity of the HACS, hydro-micro-HACS, and composite.

Sample	a*	b*	L*	Particle Size/μm	Viscosity/cP	Weight Loss Rate/%	Decomposition Temperature/°C	Crystallinity/%
HACS	−1.23 ± 0.01 a	9.32 ± 0.01 b	97.02 ± 1.01 a	12.52 ± 1.06 b	362 ± 1.00 a	80.99 ± 1.11 a	316.98 ± 2.08 b	20.87 ± 0.15 a
Hydro-micro-HACS	−1.24 ± 0.01 a	9.33 ± 0.02 b	97.03 ± 0.99 a	12.68 ± 0.01 b	270 ± 3.00 b	80.29 ± 0.50 a	317.84 ± 1.09 a	16.98 ± 0.23 b
Composite	−1.24 ± 0.02 a	20.90 ± 3.01 a	94.10 ± 2.02 b	27.65 ± 2.96 a	234 ± 3.00 c	74.83 ± 2.30 b	309.41 ± 2.10 c	12.11 ± 0.12 c

Mean ± SD values. Different lowercase letters after the number in the same column indicate a significant difference (*p* < 0.05). a*, redness; b*, yellowness; L*, lightness.

**Table 2:** DSC data of the HACS, hydro-micro-HACS, and composite.

Sample	Peak1				Peak2			
	To/°C	Tp/°C	Tc/°C	∆H (J/g)	To/°C	Tp/°C	Tc/°C	∆H (J/g)
HACS	-	-	-	-	132.95 ± 0.10 b	133.05 ± 0.15 b	139.16 ± 0.11 a	13.34 ± 0.10 a
Hydro-micro-HACS	-	-	-	-	134.78 ± 0.18 a	135.39 ± 0.10 a	138.61 ± 0.09 b	6.78 ± 0.12 b
Composite	84.77 ± 0.23	95.79 ± 0.21	104.97 ± 0.14	6.91 ± 0.20	134.76 ± 0.04 a	135.23 ± 0.12 a	138.62 ± 0.02 b	5.65 ± 0.34 c

Mean ± SD values. Different lowercase letters following the numbers in the same column indicate significant differences (*p* < 0.05).

## Data Availability

The data presented in this study are available on request from the corresponding author.
